# Replication of the Association Between Keratoconus and Polymorphisms in *PNPLA2* and *MAML2* in a Han Chinese Population

**DOI:** 10.3389/fgene.2020.00827

**Published:** 2020-07-22

**Authors:** Jing Zhang, Yue Li, Yiqin Dai, Jianjiang Xu

**Affiliations:** ^1^Eye Institute and Department of Ophthalmology, Eye & ENT Hospital, Fudan University, Shanghai, China; ^2^NHC Key Laboratory of Myopia, Fudan University, Shanghai, China; ^3^Shanghai Key Laboratory of Visual Impairment and Restoration, Shanghai, China

**Keywords:** keratoconus, association study, Han Chinese population, SNP, replication

## Abstract

Keratoconus (KC) is a complex ocular disease that is affected by both genetic and non-genetic triggers. A recent genome-wide association study (GWAS) identified a genome-wide significant locus for KC in the region of *PNPLA2* (rs61876744), as well as a suggestive signal in the *MAML2* (rs10831500) locus. In order to validate their findings, here we performed a replication study of the Han Chinese population, with 120 sporadic KC cases and 206 gender and age matched control subjects, utilizing the TaqMan SNP genotyping assays. SNP rs10831500, as well as two proxy SNPs for rs61876744, named rs7942159 and rs28633403, were subjected to genotyping. However, we did not find a significant difference (*P* > 0.05) in all the three genotyped SNPs between KC cases and the controls. A further meta-analysis on four previous cohorts of white patients and this Han Chinese cohort showed a significant genetic heterogeneity within the replicated loci. Thus, the current study suggests that SNP rs61876744 (or its proxy SNPs) and rs10831500 might not be associated with KC susceptibility in this Han Chinese cohort, and a large-scale association analysis focusing on the loci is therefore warranted in further investigations.

## Introduction

Keratoconus (KC) is a degenerative ocular disorder that is characterized by continuous corneal thinning and steepening, which finally causes moderate to severe visual impairment (Rabinowitz, 1998). Most of these diagnosed cases are sporadic, while a familial form of KC is also observed. The prevalence of KC has been estimated to be 1:2,000 in the general population. However, a strikingly higher incidence among Asians has been reported, and Asians are younger at presentation and require corneal grafting at an earlier age. This is suggestive of substantial influences of ethnic differences underlying this disease (Kok et al., 2012). The therapeutic intervention of KC varies heavily on the clinical stage. Contact lenses and corneal collagen UV cross-linking are major effective approaches for the management of KC at early stages, achieving biomechanical stabilization of the cornea and reducing the disease progression rate (Karolak and Gajecka, 2017). Unfortunately, not all KC cases are recognized at early stages, and as the disease progresses, corneal transplantation is necessitated for up to 20% of KC patients. KC is therefore one of the major indications for corneal transplantation in western countries (Faria-Correia et al., 2015). This makes finding specific biomarkers that can target KC at its early stage of particular importance.

KC has a complicated etiology, with UV exposure (Arnal et al., 2011), atopy (Bawazeer et al., 2000), contact lens wear (Steahly, 1978), and constant eye rubbing (McMonnies, 2009) considered as the main behavioral and environmental risk factors for the disease. Biologically, down-regulation of collagens and structural proteins like lumican, keratocan, and decorin, as well as increased expression of catabolic enzymes were observed in KC patients, indicating the dramatic rearrangement of the corneal architecture (Sharif et al., 2018; Ferrari and Rama, 2020). Altered TGF-β signaling, which is a key regulator of extracellular matrix (ECM) secretion and assembly, was found to be involved in KC progression (Engler et al., 2011). In addition, increased oxidative stress and classic pro-inflammatory proteins including IL1, IL6, MMP9, and TNF-α were also found in KC corneas (Mas Tur et al., 2017; Vallabh et al., 2017). More importantly, an increasing body of evidence suggests a substantial genetic basis underlying KC, such as the increased probability for siblings of KC to develop the same disease (Naderan et al., 2016), the higher concordance rate in monozygotic twins compared to dizygotic twins (Tuft et al., 2012), and the observation of multi-generation pedigrees with KC (Burdon and Vincent, 2013). Many efforts have therefore been made to identify the genetic risks for KC, mainly based on approaches including linkage analyses and genome-wide association studies (GWAS). To date, single nucleotide polymorphism (SNPs) in these genes have been identified, including *CAST, RAB3GAP1, DOCK9, LOX, HGF, ZNF469, VSX1, IL1A, IL1B, WNT10A, SOD1* (De Bonis et al., 2011; Bykhovskaya et al., 2012; Czugala et al., 2012; Li et al., 2012, 2013a,b; Wang et al., 2013; Cuellar-Partida et al., 2015), and some central corneal thickness (CCT) related loci including *MDPZ-NF1B, FOXO1, FND3B, COL4A3, COL4A4*, and *COL5A* (Lu et al., 2013; Iglesias et al., 2018). Several of them were independently investigated in other ethnicities, including the Han Chinese population, whilst substantial heterogeneity remains across various ethnicities (Wang et al., 2013, 2016, 2018; Hao et al., 2015; Zhang et al., 2018).

Recently, McComish et al. performed a GWAS study of four independent cohorts of white patients with KC. Two novel loci showed genome-wide significance, rs61876744 in the *PNPLA2* gene on chr11, and rs138380 in the *CSNK1E* gene on chr22. They also reported a suggestive association signal from rs10831500, which was close to the *MAML2* gene on chr11 (McComish et al., 2019). However, given the potential genetic heterogeneity underlying KC etiology, it still remains unclear whether these newly identified SNPs are still in association with KC risk in other populations. An intensive investigation on the loci of interest, is therefore in demand. We thus conducted a replication study here to examine their roles in KC susceptibility in an independent Han Chinese cohort.

## Materials and Methods

### Subjects

A total of 120 sporadic Han Chinese keratoconus cases, as well as 206 age and gender matched controls were recruited. KC cases were collected from the Department of Ophthalmology at the EENT Hospital of Fudan University from October 2015 to March 2018. They all lived in East China and were of Han Chinese ethnicity. KC cases were diagnosed based on both clinical examination and videokeratography pattern analysis, according to the following criteria: (1) at least one KC sign by slit-lamp examination (stromal thinning, Fleischer's ring, Munson's sign, and Vogt's striae); (2) an asymmetric bowtie pattern in corneal topography; refractive errors; signs of videokeratography; (3) KISA index >100; central K reading >47D. The control subjects had no ocular disease and attended the same hospital due to accidental injury. Written informed consent forms were signed by all participants. This study was performed in accordance with the declaration of Helsinki and was approved by the Ethics Committee of the EENT Hospital of Fudan University.

### DNA Extraction

Genomic DNA was extracted from the monocytes in peripheral blood, with the QIAGEN FlexiGene DNA kit (Qiagen, Germany) following the standard protocol. DNA concentration was tested by a NanoDrop spectrophotometer. DNA samples were stored at −20°C before use.

### SNP Genotyping

SNP rs10831500, as well as two proxy SNPs for rs61876744, named rs7942159 and rs28633403 were subjected to genotyping. The probes were designed by ThermoFisher TaqMan™ SNP genotyping Assay (Catalog nos. C__30938976_10 for rs10831500, C__11279798_10 for rs7942159, C__64236579_10 for rs28633403). The probe for SNP rs138380 failed to be designed by the custom TaqMan™ SNP genotyping Assay, and it was not further investigated here. Real-time PCR (Applied Biosystems VII, USA) was applied to complete the genotyping assay. Each reaction for the samples was prepared as 5 μL 2× SuperMix for SNP Genotyping (ThermoFisher, USA), 0.25 μL 40× probe, 2.5 μL ddH_2_O, and 2 μL DNA. PCR cycling conditions were 95°C for 10 min, 45 cycles of 95°C for 15 s and 60°C for 1 min. Fluorescence data were automatically analyzed by QuantStudio™ Real-Time PCR Software (Applied Biosystems, USA). Genotypes were classified by the ratio of the two fluorescence signals (FAM and VIC).

### Data Analysis

The statistical analyses were mainly carried out by PLINK (Purcell et al., 2007). The validation of SNP frequency in cases and controls was calculated for departure from the Hardy-Weinberg equilibrium through an exact test. The allele frequency of each SNP between the cases and controls was calculated with a χ^2^*-*test. The logistic regression model, with adjustment for gender and age, was applied to evaluate odds ratios (ORs) and their 95% confidence intervals (CIs). The linkage disequilibrium (LD) among SNPs was calculated using the LDlink package (Machiela and Chanock, 2015). A meta-analysis was performed by weighting effect size estimates using the inverse of the corresponding standard errors. The between-study heterogeneity was evaluated by the *I*^2^-value. OR and 95% CI for the minor allele were calculated with the random effects model when *I*^2^ > 50%. The statistical significance of SNP association was calculated by the *Z*-test. The *P*-values were transformed from the *Z*-scores and a pooled *P* < 0.05 was considered as statistically significant.

## Results

A total of 120 sporadic Han Chinese KC cases and 206 controls were recruited for this study. As presented in Table 1, KC cases showed an average age of 22.77 ± 5.69 yrs, and 75.8% of them were male. The control subjects showed an average age of 26.23 ± 4.17 yrs, and the percentage of males was 61.6%, similar to that of the case group.

**Table 1:** Characteristics of KC cases and controls included in this study.

**Feature**	**Cases (*n* = 120)**	**Controls (*n* = 206)**
Gender (female/male)	29/91	79/127
Average age (years)^*^	22.77 ± 5.69	26.23 ± 4.17
Age range (years)	13–45	15–33
Disease onset age (years)^*^	20.96 ± 5.08	NA
Visual activity^*^	OS: 0.61 ± 0.25	NA
	OD: 0.35 ± 0.26	

*OS, left eye; OD, right eye*.

* *Data is shown as mean ± S.D*.

**Figure 1:**
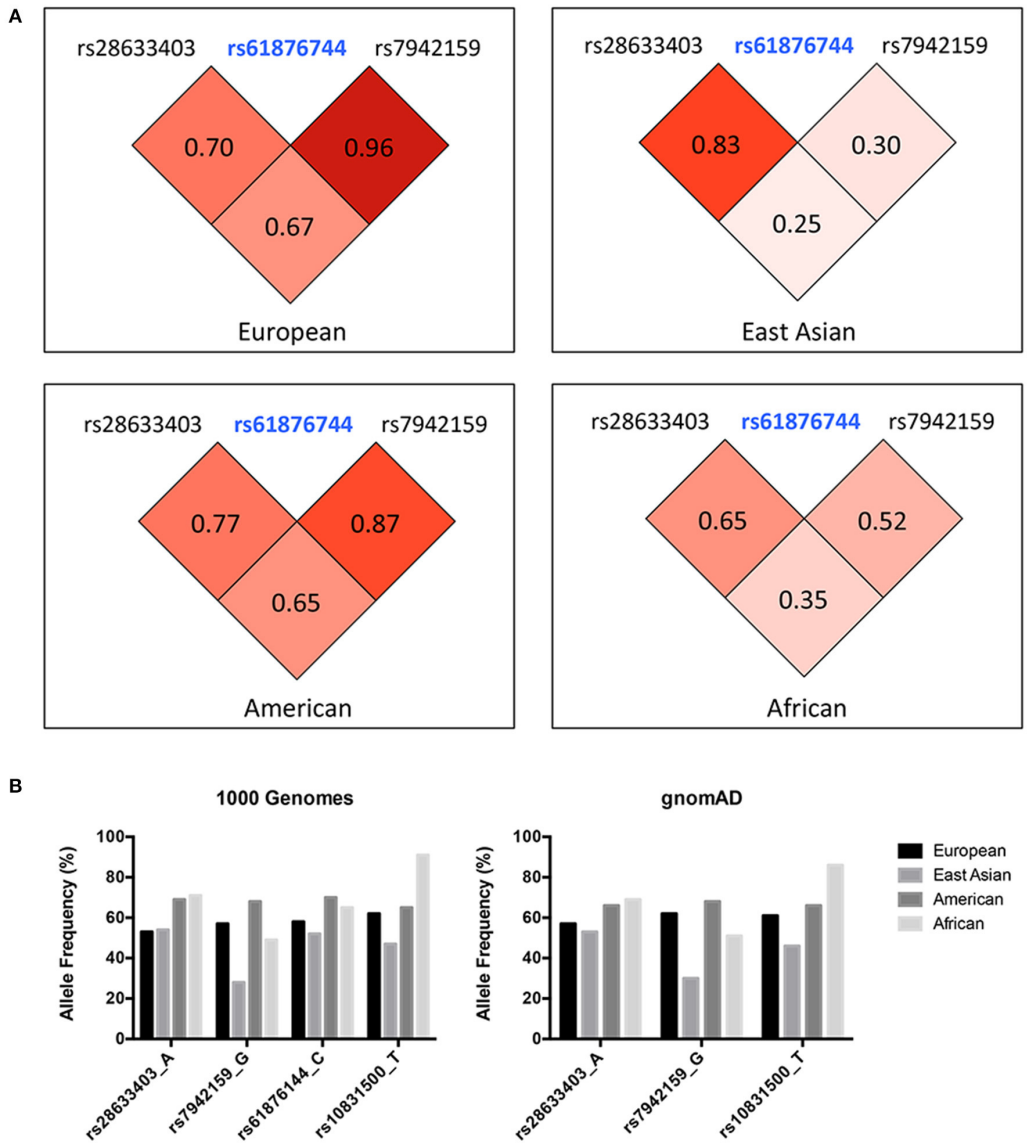
**(A)** LD pattern of SNP rs61876744 and its two proxy SNPs in the *PNPLA2* gene (shown as pairwis*e r*^2^ values in Europeans, East Asians, Americans, and Africans. Data was obtained from the 1000G Project Phase 3). **(B)** Allele frequencies of the investigated SNPs among different ancestries. Data was retrieved from the 1000 Genomes project and the gnomAD database (Allele frequency for SNP rs61876144 was not available in gnomAD).

Three SNPs were subjected to genotyping in our cohort. SNP rs10831500 in the *MAML2* gene was directly replicated here to investigate its association in this Han Chinese cohort. SNP rs61876744 in the *PNPLA2* gene showed the most significant association signal in the original GWAS, however, the TaqMan probe for this SNP failed to be designed, probably due to the features of flanking sequences around this SNP, and thereby its two proxy SNPs, rs28633403 (the most correlated SNP in Asians, *r*^2^ = 0.83) and rs7942159 (the most correlated SNP in Europeans, *r*^2^ = 0.95) were selected for further replication. The LD pattern among the three SNPs in the *PNPLA2* region, and their allele frequencies varied a lot in different ancestries (shown in Figure 1). Another suggestive signal in the *CSNK1E* gene, SNP rs138378, was not further replicated due to the failure of designing its custom probe for genotyping, as well as the lack of suitable proxy SNP (*r*^2^> 0.8).

We achieved an averaged genotyping call rate of 92.9% for the investigated SNPs. The two proxy SNPs for rs61876744 were in Hardy-Weinberg equilibrium in the controls, whilst SNP rs10831500 showed a slight deviation (*P* = 0.02905). Allelic association analyzed by PLINK showed that none of the SNPs were significantly in association with KC susceptibility in this Han Chinese cohort (Table 2). The minor allele frequency (MAF) of rs28633403 in the case group was almost comparable to that in the control group (49.4 vs. 50.0%). SNP rs7942159, the other proxy SNP for rs61876744 showed a 5% MAF difference between the cases and the controls, but did not reach nominal significance. Interestingly, its risk allele “G” had much lower frequency in Asians (Asians: 30%, Europeans: 61.5%; gnomAD data). For SNP rs10832500, its protective allele “T” in the original GWAS, presented a risk role in this Han Chinese cohort. A following genotypic association analysis was performed. However, only the genotype distribution of rs7942159 presented a borderline difference (*P* = 0.06726). The frequencies of the GG, GA, and AA genotypes of rs7942159 were found to be 8.2, 53.4, and 38.4% in the KC case group, compared to 11.1, 37.7, and 51.2% in the control group. A higher OR of 1.69 was shown when the dominant model was applied (Table 3).

**Table 2:** Basic association result of the genotyped SNPs in this study.

**SNP**	**Allele**	**MAF_**	**MAF_**	**χ^**2**^**	***P*-value**	**OR (95% CI)**
		**Case %**	**Control %**			
rs28633403	A/G	49.4	50.0	0.01639	0.8981	0.98 (0.67–1.42)
rs7942159	G/A	34.9	29.9	1.163	0.2808	1.26 (0.83–1.91)
rs10831500	T/G	51.4	45.8	1.716	0.1901	1.25 (0.89–1.74)

*MAF, minor allele frequency, the minor allele of each SNP was underlined; OR, odds ratio, with respect to the minor allele; 95% CI: Lower/Upper bound of 95% confidence interval for OR; P-values and ORs were calculated after adjustment for age and gender*.

**Table 3:** Genotype frequencies of the genotyped SNPs and their association with susceptibility to KC.

**SNP/group**	**Group frequency**			***P*-value**	**Dominant model**		**Recessive model**	
					**OR (95% CI)**	***P*-value**	**OR (95% CI)**	***P*-value**
rs28633403	AA	AG	GG	0.5483	AA&AG vs. GG		AA vs. GG&AG	
Cases	20.7%	57.3%	22.0%		1.18 (0.63–2.22)	0.5962	0.78 (0.41–1.49)	0.4556
Controls	25.0%	50.0%	25.0%					
rs7942159	GG	GA	AA	0.07729	GG&GA vs. AA		GG vs. AA&GA	
Cases	8.2%	53.4%	38.4%		1.69 (0.96–2.97)	0.06726	0.72 (0.27–1.89)	0.4981
Controls	11.1%	37.7%	51.2%					
rs10831500	TT	TG	GG	0.3785	TT&TG vs. GG		TT vs. GG&TG	
Cases	32.4%	37.8%	29.7%		1.18 (0.71–1.96)	0.5171	1.18 (0.71–1.96)	0.5171
Controls	25.0%	41.7%	33.3%					

*OR, odds ratio; CI, confidence interval; P-values and ORs were calculated after adjustment for age and gender*.

**Figure 2:**
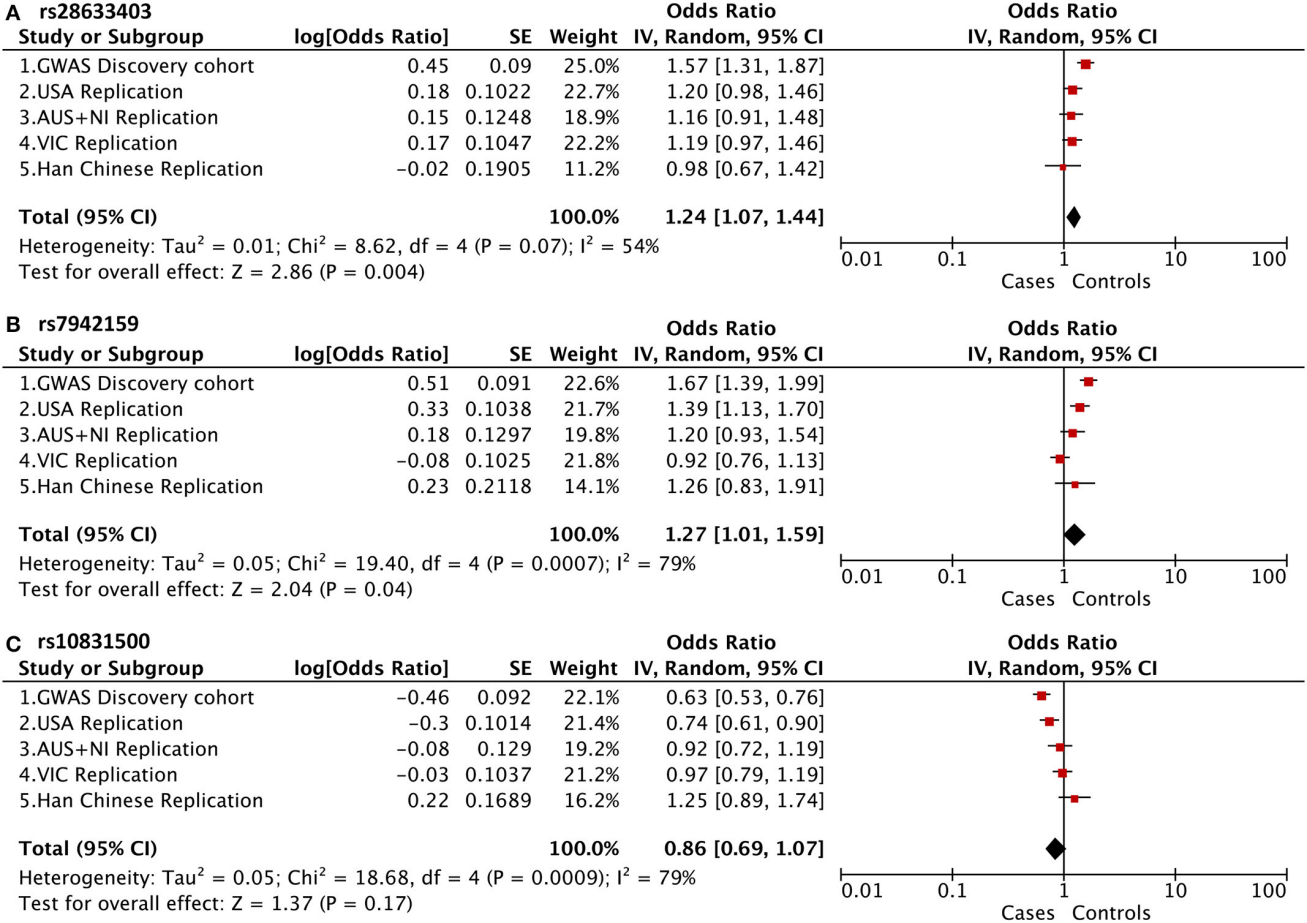
Meta-analysis of association results from previous four cohorts of white patients and this Han Chinese cohort, shown as a forest plot of the three genotyped SNPs. **(A)** *PNPLA2* rs28633403; **(B)** *PNPLA2* rs7942159; **(C)** *MAML2* rs10831500. The size of the box is proportional to the weight of the study. Squares indicate the study-specific odds ratio (OR). Horizontal lines indicate 95% confidence interval (CI). A diamond shows the summary OR with its corresponding 95% CI. USA, United States; AUS+NI, Australia and Northern Ireland; VIC, Victoria, Australia.

Of note, in addition to rs10832500, SNP rs28633403, and rs7942159 were also genotyped in the original GWAS project, and the raw summary data was obtained (Supplementary Table 1). A meta-analysis of association results from previous four cohorts of white patients and this Han Chinese cohort was then further performed (Figure 2). It was found that these SNPs presented opposite trends among the included five cohorts, and substantial between-study heterogeneity was found. Therefore, the random-effects model was used here. SNP rs28633403 and rs7942159 were found to be in association with KC by meta-analysis (*P_meta* = 0.004 and 0.04, respectively). However, their contributions to KC susceptibility remain questionable, as substantial heterogeneity existed (*I*^2^ > 50%) and their association *P*-values in 3 out of 5 cohorts were bigger than the 0.05 cutoff. SNP rs10832500 did not show significant association with KC by meta-analysis. Taken together, due to the substantial heterogeneity within the replicated loci, the current study did not support the association between KC and SNPs in *PNPLA2* and *MAML2* in this Han Chinese cohort.

## Discussion

The etiology of KC is not well-understood, with genetic, environmental, and behavioral risk factors all contributing to the disease. Identifying the genetic risk factors for KC has proved challenging. Recently, well-powered GWAS for keratoconus and central corneal thickness have uncovered many risk loci, but most of them were performed in western populations (Burdon et al., 2011; Li et al., 2012; Lu et al., 2013; Cuellar-Partida et al., 2015; Khawaja et al., 2019; McComish et al., 2019). Some of those reported KC susceptibility loci have been further investigated in a Han Chinese cohort, including by our group (Wang et al., 2013, 2018; Hao et al., 2015; Zhang et al., 2018). However, not all these established KC-associated loci could be successfully validated, highlighting the great genetic heterogeneity underlying this complicated disease between Asians and Europeans.

Here we replicated the association of SNPs in the *PNPLA2* and *MAML2* gene with KC susceptibility in a Han Chinese cohort. We were unable to discover a remarkable difference (*P* > 0.05) in all the three genotyped SNPs between KC cases and the controls. Further meta-analysis on previous four cohorts of white patients and this Han Chinese cohort showed a significant genetic heterogeneity within the replicated loci. Thus, the current study suggested that SNP rs61876744 (or its proxy SNPs) and rs10831500 might not link with KC susceptibility in this Han Chinese cohort. Actually, based on the original GWAS, only rs61876744 was selected to represent the association signal of this locus due to its qualified *P*-value (*P* < 5 × 10^−8^) and the same direction of association among the four examined white cohorts. It is also possible that other SNPs within the *PNPLA2* locus may confer the risk to KC susceptibility, and thereby a large-scale association analysis on other candidate SNPs is required in further investigations.

The current study indicated great heterogeneity within the *PNPLA2* and *MAML2* region, as the *I*^2^*-*values calculated by the meta-analysis for all these investigated SNPs were larger than 50%. The discrepancy between original GWAS and the meta-analysis results might come from the existence of false positive signals from GWAS, and more likely, could be explained by their substantial population differences across various ancestries. Indeed, the allele frequency (AF) of these SNPs, as well as the LD patterns within, varied a lot among different populations (Figure 1B). For SNP rs7942159, which was in high LD (*r*^2^ = 0.96) with rs61876144, the lead SNP in previous GWAS in Europeans, showed a markedly reduced AF in East Asians (57 vs. 28%). Consistently, its LD (shown as *r*^2^) with rs61876144 reduced to 0.30 in East Asians. The heterogeneity *P*-value for rs7942159 in the meta-analysis on four white cohorts and this Chinese cohort was 0.0007. The “G” allele of rs7942159 was the risk allele in both Europeans and East Asians, although the “G” allele is the minor allele in East Asians, but major allele in Europeans. Similarly, the “A” allele of rs28633403 was the risk allele for both populations, while its AF differed a lot. For SNP rs10831500 (*MAML2* locus), replication in the Han Chinese cohort and the subsequent meta-analysis did not support its association to KC susceptibility. Actually, in the original GWAS, the signal from rs10831500 was supported by the US replication cohort only. Its association *P*-values in another two white cohorts were both larger than 0.5. More interestingly, its risk allele was even contradictory in the Han Chinese cohort, making the causative role of rs10831500 to KC susceptibility questionable.

This study had several limitations that need to be noted. The primary limitation came from the relatively small sample size here, which might cause lower power and negative findings. We suggested that SNP rs28633403 and rs10831500 should not be associated to KC in Han Chinese, due to their similar allele frequencies in KC cases and controls, or the contrasting risk allele among different cohorts. However, the contribution of rs7942159 to KC risk is worth further exploration with an increased sample size, although the dominant allele differed among ethnicities. The association of other outstanding SNPs in the *PNPLA2* also needs attention. Secondly, due to the failure to design suitable probes for direct genotyping on the lead SNP in previous GWAS, two proxy SNPs for rs61876144 were genotyped instead. We speculated that the failure of designing suitable probes might be due to the features of the flanking sequences around rs61876144, as they may affect the efficiency or specificity of PCR amplification reactions. Although we have already selected the most correlated proxy SNPs for replication instead, they were not in absolute LD with the lead SNP, and this might influence the outcomes.

In conclusion, this case-control study of a Han Chinese cohort did not support the association of SNPs in the *PNPLA2* and *MAML2* gene and KC susceptibility, which was suggested by a previous GWAS report. Nevertheless, we could not fully rule out the probability that other SNPs within the loci might contribute to KC risk. Further investigations are required to explore other potential causative variants within the loci.

## Data Availability Statement

All data relevant to the study are included in the article/Supplementary Material.

## Ethics Statement

The studies involving human participants were reviewed and approved by the Ethics Committee of Eye and ENT Hospital of Fudan University. The patients/participants provided their written informed consent to participate in this study.

## Author Contributions

JX and JZ designed the study. JZ, YL, and YD performed the experiments. JZ analyzed the data. JZ, YL, YD, and JX wrote and revised the manuscript. All authors contributed to the article and approved the submitted version.

## Conflict of Interest

The authors declare that the research was conducted in the absence of any commercial or financial relationships that could be construed as a potential conflict of interest.

## References

[B1] Arnal E. Peris-Martinez C. Menezo J. L. Johnsen-Soriano S. Romero F. J. (2011). Oxidative stress in keratoconus? Invest. Ophthalmol. Visual Sci. 52, 8592–8597. 10.1167/iovs.11-7732 21969298

[B2] Bawazeer A. M. Hodge W. G. Lorimer B. (2000). Atopy and keratoconus: a multivariate analysis. Br. J. Ophthalmol. 84, 834–836. 10.1136/bjo.84.8.834 10906086 PMC1723585

[B3] Burdon K. P. Macgregor S. Bykhovskaya Y. Javadiyan S. Li X. Laurie K. J.. (2011). Association of polymorphisms in the hepatocyte growth factor gene promoter with keratoconus. Invest. Ophthal. Vis. Sci. 52, 8514–8519. 10.1167/iovs.11-8261 22003120 PMC3208191

[B4] Burdon K. P. Vincent A. L. (2013). Insights into keratoconus from a genetic perspective. Clin. Exp. Optometry 96, 146–154. 10.1111/cxo.12024 23387289

[B5] Bykhovskaya Y. Li X. Epifantseva I. Haritunians T. Siscovick D. Aldave A.. (2012). Variation in the lysyl oxidase (LOX) gene is associated with keratoconus in family-based and case-control studies. Investig. Ophthalmol. Visual Sci. 53, 4152–4157. 10.1167/iovs.11-9268 22661479 PMC3760233

[B6] Cuellar-Partida G. Springelkamp H. Lucas S. E. Yazar S. Hewitt A. W. Iglesias A. I.. (2015). WNT10A exonic variant increases the risk of keratoconus by decreasing corneal thickness. Hum. Mol. Genet. 24, 5060–5068. 10.1093/hmg/ddv211 26049155

[B7] Czugala M. Karolak J. A. Nowak D. M. Polakowski P. Pitarque J. Molinari A.. (2012). Novel mutation and three other sequence variants segregating with phenotype at keratoconus 13q32 susceptibility locus. Eur. J. Hum. Genet. 20, 389–397. 10.1038/ejhg.2011.203 22045297 PMC3306853

[B8] De Bonis P. Laborante A. Pizzicoli C. Stallone R. Barbano R. Longo C.. (2011). Mutational screening of VSX1, SPARC, SOD1, LOX, and TIMP3 in keratoconus. Mol. Vision 17, 2482–2494. 21976959 PMC3185016

[B9] Engler C. Chakravarti S. Doyle J. Eberhart C. G. Meng H. Stark W. J.. (2011). Transforming growth factor-beta signaling pathway activation in Keratoconus. Am. J. Ophthalmol. 151, 752–9 e2. 10.1016/j.ajo.2010.11.008 21310385 PMC3079764

[B10] Faria-Correia F. Luz A. Ambrosio R. (2015). Managing corneal ectasia prior to keratoplasty. Expert. Rev. Ophthalmol. 10, 33–48. 10.1586/17469899.2015.991390

[B11] Ferrari G. Rama P. (2020). The keratoconus enigma: a review with emphasis on pathogenesis. Ocular Surf. 18, 363–373. 10.1016/j.jtos.2020.03.006 32234342

[B12] Hao X. D. Chen P. Chen Z. L. Li S. X. Wang Y. (2015). Evaluating the Association between Keratoconus and Reported Genetic Loci in a Han Chinese Population. Ophthalmic genetics 36, 132–136. 10.3109/13816810.2015.1005317 25675348

[B13] Iglesias A. I. Mishra A. Vitart V. Bykhovskaya Y. Hohn R. Springelkamp H.. (2018). Cross-ancestry genome-wide association analysis of corneal thickness strengthens link between complex and Mendelian eye diseases. Nat. Commun. 9:1864. 10.1038/s41467-018-03646-6 29760442 PMC5951816

[B14] Karolak J. A. Gajecka M. (2017). Genomic strategies to understand causes of keratoconus. Mol. Genet. Genom. 292, 251–269. 10.1007/s00438-016-1283-z 28032277 PMC5357269

[B15] Khawaja A. P. Rojas Lopez K. E. Hardcastle A. J. Hammond C. J. Liskova P. Davidson A. E. (2019). Genetic variants associated with corneal biomechanical properties and potentially conferring susceptibility to keratoconus in a genome-wide association study. JAMA Ophthal. 137, 1005–1012. 10.1001/jamaophthalmol.2019.2058 PMC6604088 31246245

[B16] Kok Y. O. Tan G. F. Loon S. C. (2012). Review: keratoconus in Asia. Cornea 31, 581–93. 10.1097/ICO.0b013e31820cd61d 22314815

[B17] Li X. Bykhovskaya Y. Canedo A. L. Haritunians T. Siscovick D. Aldave A. J.. (2013b). Genetic association of COL5A1 variants in keratoconus patients suggests a complex connection between corneal thinning and keratoconus. Investig. Ophthalmol. Visual Sci. 54, 2696–2704. 10.1167/iovs.13-11601 23513063 PMC3630822

[B18] Li X. Bykhovskaya Y. Haritunians T. Siscovick D. Aldave A. Szczotka-Flynn L.. (2012). A genome-wide association study identifies a potential novel gene locus for keratoconus, one of the commonest causes for corneal transplantation in developed countries. Hum. Mol. Genet. 21, 421–429. 10.1093/hmg/ddr460 21979947 PMC3276283

[B19] Li X. Bykhovskaya Y. Tang Y. G. Picornell Y. Haritunians T. Aldave A. J.. (2013a). An association between the calpastatin (CAST) gene and keratoconus. Cornea 32, 696–701. 10.1097/ICO.0b013e3182821c1c 23449483 PMC3653445

[B20] Lu Y. Vitart V. Burdon K. P. Khor C. C. Bykhovskaya Y. Mirshahi A.. (2013). Genome-wide association analyses identify multiple loci associated with central corneal thickness and keratoconus. Nat. Genet. 45, 155–163. 10.1038/ng.2506 23291589 PMC3720123

[B21] Machiela M. J. Chanock S. J. (2015). LDlink: a web-based application for exploring population-specific haplotype structure and linking correlated alleles of possible functional variants. Bioinformatics 31, 3555–3557. 10.1093/bioinformatics/btv402 26139635 PMC4626747

[B22] Mas Tur V. MacGregor C. Jayaswal R. O'Brart D. Maycock N. (2017). A review of keratoconus: diagnosis, pathophysiology, and genetics. Survey Ophthalmol. 62, 770–783. 10.1016/j.survophthal.2017.06.009 28688894

[B23] McComish B. J. Sahebjada S. Bykhovskaya Y. Willoughby C. E. Richardson A. J. Tenen A.. (2019). Association of genetic variation with keratoconus. JAMA Ophthalmol. 138, 174–181. 10.1001/jamaophthalmol.2019.5293 31855235 PMC6990728

[B24] McMonnies C. W. (2009). Mechanisms of rubbing-related corneal trauma in keratoconus. Cornea 28, 607–615. 10.1097/ICO.0b013e318198384f 19512912

[B25] Naderan M. Rajabi M. T. Zarrinbakhsh P. Naderan M. Bakhshi A. (2016). Association between family history and keratoconus severity. Curr. Eye Res. 41, 1414–1418. 10.3109/02713683.2015.1128553 27158890

[B26] Purcell S. Neale B. Todd-Brown K. Thomas L. Ferreira M. A. Bender D.. (2007). PLINK: a tool set for whole-genome association and population-based linkage analyses. Am. J. Hum. Genet. 81, 559–575. 10.1086/519795 17701901 PMC1950838

[B27] Rabinowitz Y. S. (1998). Survey of ophthalmology. Keratoconus 42, 297–319. 10.1016/S0039-6257(97)00119-7 9493273

[B28] Sharif R. Bak-Nielsen S. Hjortdal J. Karamichos D. (2018). Pathogenesis of Keratoconus: the intriguing therapeutic potential of Prolactin-inducible protein. Progress Retinal Eye Res. 67, 150–167. 10.1016/j.preteyeres.2018.05.002 29758268 PMC6235698

[B29] Steahly L. P. (1978). Keratoconus following contact lens wear. Ann. Ophthalmol. 10, 1177–1179. 736404

[B30] Tuft S. J. Hassan H. George S. Frazer D. G. Willoughby C. E. Liskova P. (2012). Keratoconus in 18 pairs of twins. Acta Ophthalmol. 90, e482–e486. 10.1111/j.1755-3768.2012.02448.x 22682160

[B31] Vallabh N. A. Romano V. Willoughby C. E. (2017). Mitochondrial dysfunction and oxidative stress in corneal disease. Mitochondrion 36, 103–113. 10.1016/j.mito.2017.05.009 28549842

[B32] Wang Y. Jin T. Zhang X. Wei W. Cui Y. Geng T.. (2013). Common single nucleotide polymorphisms and keratoconus in the Han Chinese population. Ophthalmic Genet. 34, 160–166. 10.3109/13816810.2012.743569 23289806

[B33] Wang Y. Wei W. Zhang C. Zhang X. Liu M. Zhu X.. (2016). Association of Interleukin-1 gene single nucleotide polymorphisms with keratoconus in Chinese Han Population. Curr. Eye Res. 41, 630–635. 10.3109/02713683.2015.1045083 26200829

[B34] Wang Y. M. Ma L. Lu S. Y. Chan T. C. Y. Yam J. C. S. Tang S. M.. (2018). Analysis of multiple genetic loci reveals MPDZ-NF1B rs1324183 as a putative genetic marker for keratoconus. Br. J. Ophthalmol. 102, 1736–1741. 10.1136/bjophthalmol-2018-312218 30002070

[B35] Zhang J. Wu D. Li Y. Fan Y. Chen H. Xu J. (2018). Evaluating the association between calpastatin (CAST) gene and keratoconus in the Han Chinese population. Gene 653, 10–13. 10.1016/j.gene.2018.02.016 29428799

